# Suspensions of Medical Journals

**Published:** 1859-07

**Authors:** 


*Suspensions of Medical Journals*—We regret to be called upon to record the recent stoppage of several medical journals.

The New Hampshire Journal, a small but interesting publication, has been discontinued. The Maine Medical and Surgical Reporter has also stopped, as the editor implies, from want of remuneration. This was issued but a single year. The Philadelphia Medical and Surgical Journal we have not seen for a long time, and see by a notice in the Nashville Journal, that it has ceased to exist. The Montreal Medical Chemical, which is the only medical journal we have ever seen published in Canada, is now dropped, after having been published for six years. The Louisville Medical Gazette is suspended, on account of the ill health of the editor, Dr. L. J. Frazee. The Veterinary Journal, published at Boston, fills up our list of deaths. We hope that this epidemic will carry off no more of our old friends.

